# F-Spondin Is the Signal by Which 2-Methoxyestradiol Induces Apoptosis in the Endometrial Cancer Cell Line Ishikawa

**DOI:** 10.3390/ijms20163850

**Published:** 2019-08-07

**Authors:** Ramiro Rincón-Rodriguez, Dennise Mena, Javier Mena, Patricia Díaz-Saldivar, Emanuel Guajardo-Correa, Carlos Godoy-Guzman, Hugo Cardenas, Pedro A. Orihuela

**Affiliations:** 1 Facultad de Odontología, Universidad de Antioquia, Medellín 050010, Colombia; 2 Laboratorio de Inmunología de la Reproducción, Facultad de Química y Biología, Universidad de Santiago de Chile, Santiago 9160000, Chile; 3 Centro para el Desarrollo en Nanociencia y Nanotecnología-CEDENNA, Santiago 9160000, Chile; 4 Facultad de Medicina, Universidad de Santiago de Chile, Santiago 9160000, Chile

**Keywords:** cancer, 2-methoxyestradiol, F-spondin, Ishikawa cells, apoptosis

## Abstract

The metabolite 2-methoxyestradiol (2ME) is an endogenous estrogen metabolite with potential therapeutic properties in reproductive cancers. However, the molecular mechanisms by which 2ME exerts its anticancer activity are not well elucidated. The purpose of this study was to determine the molecular signals associated with the apoptotic effects of 2ME in a human endometrial cancer cell line. Ishikawa cells were treated with non-apoptotic (0.1 µM) or apoptotic concentrations (5 µM) of 2ME, and 12 hours later mRNA levels for *Scd2*, *Snx6*, and *Spon1* were determined by real-time PCR. We then investigated by immunofluorescence and Western blot the expression and distribution of F-spondin, encoded by *Spon1*, in Ishikawa cells treated with 2ME 5 µM at 6, 12, or 24 h after treatment. The role of estrogen receptors (ER) in the effect of 2ME on the *Spon1* level was also investigated. Finally, we examined whether 2ME 5 µM induces cell death in Ishikawa cells pre-incubated with a neutralizing F-spondin antibody. Non-apoptotic or apoptotic concentrations of 2ME decreased *Scd2* and increased *Snx6*. However, *Spon1* was only increased with the 2ME apoptotic concentration. F-spondin protein was also increased at 12 and 24 h after 2ME treatment, while 2ME-induced *Spon1* increase was independent of ER. Neutralization of F-spondin blocked the effect of 2ME on the cell viability. These results show that F-spondin signaling is one of the components in the apoptotic effects of 2ME on Ishikawa cells and provide experimental evidence underlying the mechanism of action of this estrogen metabolite on cancer cells.

## 1. Introduction

The metabolite 2-methoxyestradiol (2ME) is a natural metabolite of estradiol (E_2_) generated by sequential hydroxylation and methylation of estrogens [1,2]. The main steps involve the conversion of E_2_ to 2-hydroxyestradiol (2OHE_2_) and subsequent generation of 2ME. These reactions include a first oxidation in the carbon 2 inside the aromatic A-ring of E_2_ that is catalyzed by the cytochrome P450 isoform 1A1 to generate 2OHE_2_; the hydroxyl group previously added to 2OHE_2_ is replaced by a methyl group via conjugation reaction catalyzed by catechol-O-methyltransferase, generating a molecule of 2ME [3]. It has also been shown that 2ME is a potent inhibitor of angiogenesis and that inhibits tumor growth [4], but it is not cytotoxic to quiescent cells [5]. Particularly, 2ME is able to inhibit proliferation of a variety of malignant cells, an effect that differs from another estrogen metabolites [5]. 

Cancer encompassing the reproductive tract will account for 12% of all female neoplasia, and approximately 15% of mortalities are associated with this pathology [6]. In this context, endometrial cancer is the most common and with high indices of mortality in women [7]. Risk factors for endometrial cancer include obesity, diabetes mellitus, breast cancer, use of tamoxifen, nulliparity, late menopause, exposure to high levels of estrogen, and old age [8]. Chemotherapy is the main treatment for patients with endometrial cancer, although this treatment is associated with well-documented secondary effects. Thus, it is imperative to find new effective therapies for endometrial malignancy with minimal toxicity risk to normal cells. In this context, 2ME is a promissory candidate, which is now under clinical trials as a therapy for various types of cancer [1,2].

The mechanism of action of 2ME on cancer cell lines is unknown, although it is postulated that 2ME inhibits cell proliferation, blocking microtubule polymerization, and inhibiting the cell cycle [1,5]. Phosphorylation of Bcl-2 and Bcl-xL, two members of the Bcl-2 family, is disrupted by tubulin-interacting agents causing inactivation of its antiapoptotic activity [9]. Moreover, 2ME did not affect viability in tumor cells that were not in the proliferation stage, showing a selective effect of this estrogen metabolite on the machinery of the cell cycle [10]. 

A variety of human cancer cell lines have been established, contributing to elucidation of the cellular and molecular mechanisms of cancer progression in the human body, as well as the testing of new therapeutic alternatives. The Ishikawa cell line is a well differentiated human endometrial adenocarcinoma bearing estrogen and progesterone receptors so that this cell line is widely utilized in the biology of reproductive cancers [11]. It has been demonstrated that 2ME in a range of concentrations between 2 and 5 µM triggers apoptosis in the Ishikawa cells, activating both their intrinsic and extrinsic pathways [12]; furthermore, this 2ME effect does not require activation of the canonical estrogen receptors (ER), although it involves accumulation of the G2/M phase of the cell cycle previous to apoptosis triggering [12,13]. Recently, we have reported that stearoyl-coenzyme A desaturase 2 (*Scd2*), sorting nexin 6 (*Snx6*), and F-spondin (*Spon1*) are target genes of 2ME [14]. These genes are associated with intracellular traffic of membrane receptors, lipid metabolism, and extracellular matrix remodeling associated with angiogenic regulation in the mammalian uterus [14]. Thus, these genes could be useful to explore the signaling pathways of 2ME in cancer cell lines. Therefore, the purpose of this work was to determine the molecular signals associated with the apoptotic effects of 2ME in Ishikawa cells. First, we examined the expression of the 2ME-target genes *Scd2*, *Snx6*, and *Spon1* under conditions in which Ishikawa cells were exposed to non-apoptotic or apoptotic concentrations of 2ME. We reasoned that genes whose level of expression increased only in response to apoptotic concentrations of 2ME, but not to non-apoptotic concentrations, would be good candidates for further exploration. The results oriented us to investigate the role of *Spon1* and its encoded protein, F-spondin, on the apoptotic effect of 2ME in Ishikawa cells. For this, a time-course on the expression and distribution of the mRNA and F-spondin protein was investigated in Ishikawa cells treated with 2ME. The role of classical ER in the effect of 2ME on *Spon1* level was also investigated. Finally, we examined whether a neutralizing F-spondin antibody is able to block the apoptotic effect of 2ME on Ishikawa cells. 

## 2. Results

### 2.1. Spon1, but not Scd2 and Snx6, Increased with an Apoptotic Concentration of 2ME

**Figure 1:**
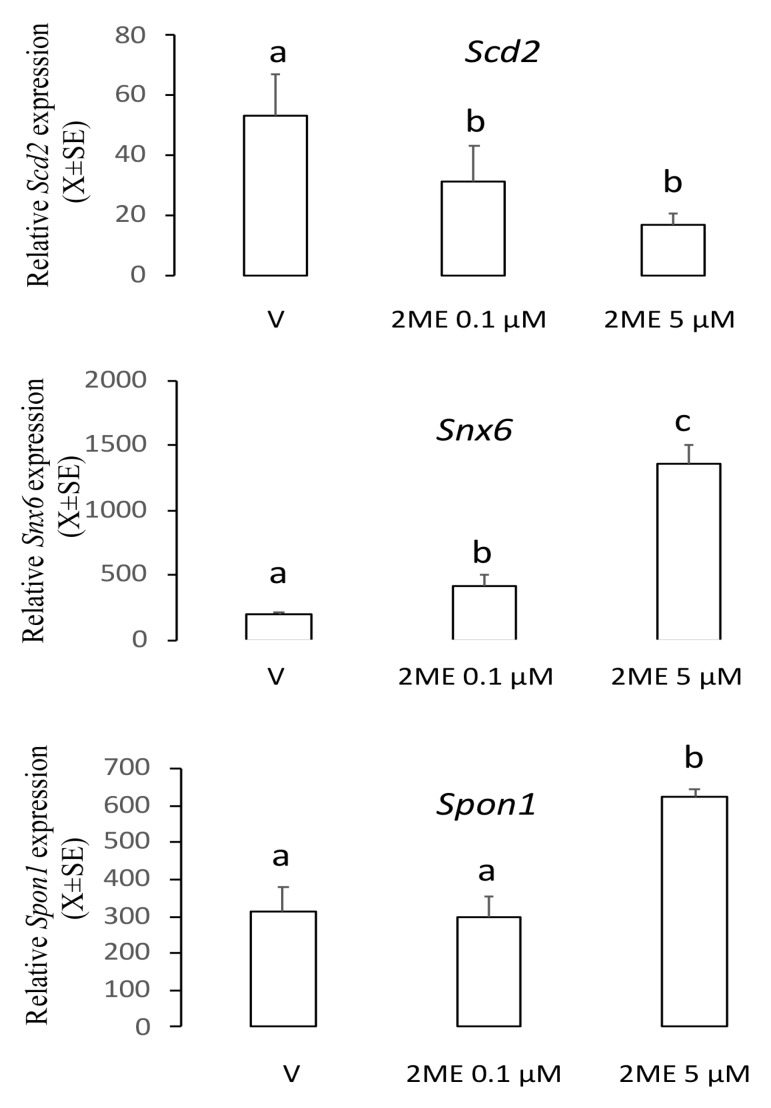
Levels of the transcripts *Scd2*, *Snx6*, or *Spon1* in the Ishikawa cells treated with a non-apoptotic (0.1 µM) or apoptotic (5 µM) concentration of 2-methoxyestradiol (2ME). Cells were incubated with 2ME for 12 h and then processed by real-time PCR. This experiment consisted of 5 replicates. a ≠ b ≠ c, *p* < 0.05. Note that *Spon1* was increased only at the apoptotic concentration of 2ME.

To determine the role of the genes *Scd2*, *Snx6*, or *Spon1* on the apoptotic effect of 2ME, we incubated Ishikawa cells with 0.1 or 5 µM of 2ME, and 12 h later cells were processed to determine mRNA levels using real-time PCR. As shown in Figure 1, analysis of the transcript levels showed that *Scd2* decreased 0.5–2 fold (V: 53.6 ± 13.8; 2ME 0.1 µM: 31.7 ± 11.9; 2ME 5 µM: 17.2 ± 3.5) and *Snx6* increased 2–6 fold (V: 196.7 ± 20.5; 2ME 0.1 µM: 417.3 ± 91.6; 2ME 5 µM: 1364 ± 133.8) in both non-apoptotic and apoptotic concentrations. However, the *Spon1* transcript increased 3-fold only with the apoptotic concentration of 2ME (V: 312.4 ± 68.4; 2ME 0.1 µM: 297.6 ± 56.6; 2ME 5 µM: 622.1 ± 21.6). Thus, we chose *Spon1* for further exploration.

### 2.2. ME Increased mRNA and Protein Levels of F-Spondin in a Time–Course Manner

**Figure 2:**
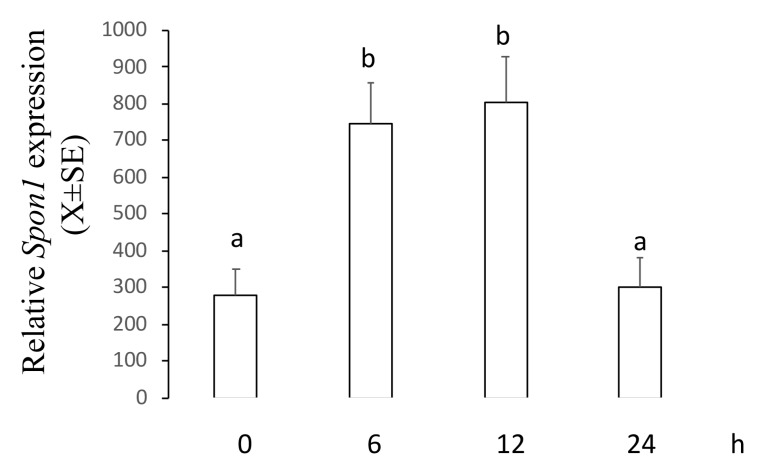
Levels of *Spon1* in the Ishikawa cells treated with 2ME 5 uM during 0, 6, 12, or 24 h and then processed by real-time PCR. This experiment consisted of 5 replicates. a ≠ b, *p* < 0.05. Note that *Spon1* was increased at 6 and 12 h after treatment.

**Figure 3:**
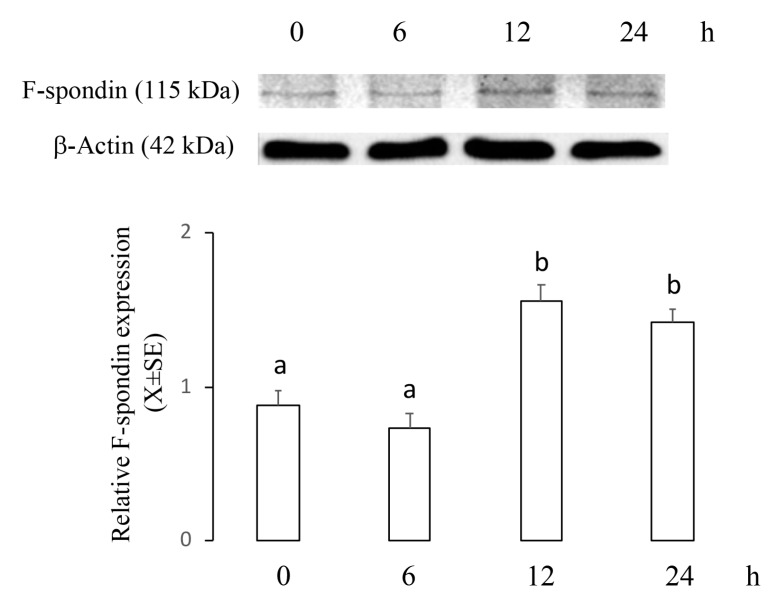
Levels of F-spondin protein in the Ishikawa cells treated with 2ME 5 μM during 0, 6, 12, or 24 h and then processed by immunoblotting followed by densitometric analysis. This experiment consisted of 5 replicates. a ≠ b, *p* < 0.05. Values were normalized to β-actin. Note that F-spondin protein was increased at 12 and 24 h after treatment.

This experiment was designed to establish whether an apoptotic concentration of 2ME increases mRNA and protein levels of F-spondin in a time–course manner. Ishikawa cells were incubated with 5 µM of 2ME, and at 0, 6, 12, or 24 h later, cells were processed to determine mRNA and protein levels using real-time PCR or Western blot. Figure 2 shows that *Spon1* transcripts increased at 6 and 12 h (0 h: 280.4 ± 70.2; 6 h: 744.3 ± 111.4; 12 h: 805 ± 121.7; 24 h: 299 ± 80.9) while Figure 3 shows that F-spondin protein increased at 12 and 24 h after 2ME treatment (0 h: 0.88 ± 0.09; 6 h: 0.73 ± 0.1; 12 h: 1.56 ± 0.1; 24 h: 1.42 ± 0.08).

### 2.3. ME Did not Change the Intracellular Distribution of F-Spondin in Ishikawa Cells

**Figure 4:**
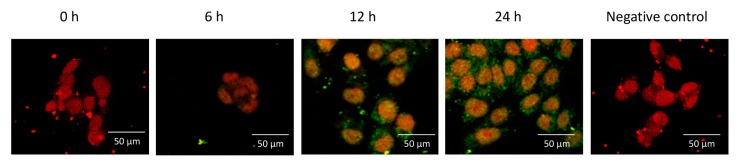
Representative photomicrographs showing the intracellular distribution of F-spondin protein (green) in the Ishikawa cells visualized by confocal microscopy. Nuclei were stained with propidium iodide (red). Note that F-spondin was mainly localized in the plasma membrane and cytoplasm after 2ME treatment. This experiment consisted of 5 replicates. Negative controls for immunoreactivity were incubated with preimmune serum.

This experiment was designed to establish if an apoptotic concentration of 2ME changes the intracellular distribution of F-spondin. Ishikawa cells were incubated with 5 µM of 2ME, and 0, 6, 12, or 24 h later, the cells were processed to determine cellular localization using confocal microscopy. Figure 4 shows that F-spondin was mainly localized in the plasma membrane and cytoplasm without any differences between treatment groups. 

### 2.4. ME-Induced Spon1 Increase Did not Require ER Activation in Ishikawa Cells

**Figure 5:**
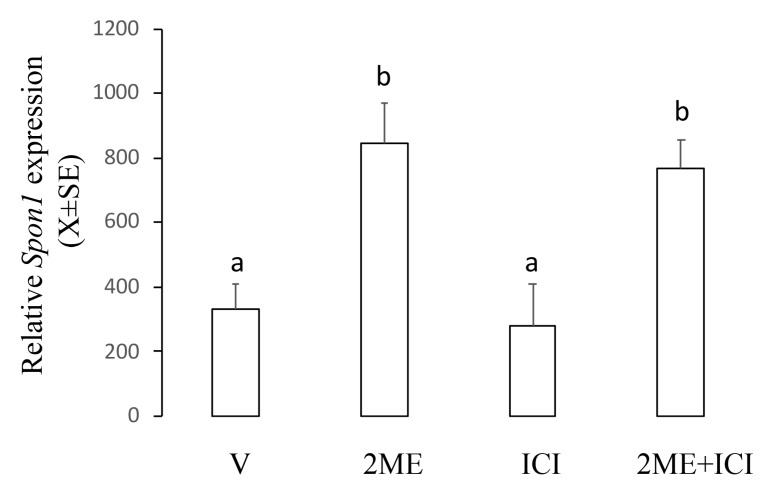
Levels of *Spon1* in the Ishikawa cells treated with 2ME alone or concomitant with the estrogen receptor antagonist ICI182780 (ICI). V is the control group. This experiment consisted of 5 replicates. a ≠ b, *p* < 0.05. Note that ICI did not affect the 2ME-induced *Spon1* increase.

This experiment was designed to establish the role of ER on the *Spon1*-increased expression induced by 2ME. For this, we used ICI182780, which inhibits activation of classical ER. Ishikawa cells were incubated with 5 µM of 2ME alone or combined with ICI182780 25 µg/mL, and 12 h later, the cells were processed to determine mRNA levels using real-time PCR. Figure 5 shows that ICI182780 alone did not affect the basal levels of *Spon1* and neither changed the 2ME-induced *Spon1* increase (V: 332.6 ± 74.9; 2ME: 845.7 ± 121.4; ICI182780: 277.7 ± 129.9; 2ME + ICI 182780: 769.5 ± 87.3).

### 2.5. Neutralizing F-Spondin Antibody Blocked the Effect of 2ME on Cell Viability

**Figure 6:**
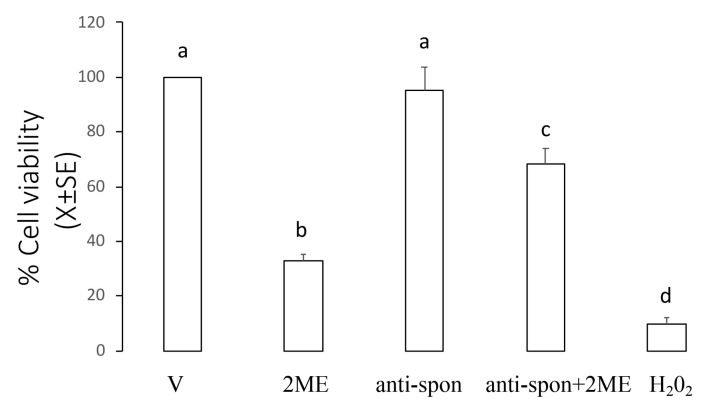
F-spondin neutralizing antibody blocked the 2ME-induced apoptosis in the Ishikawa cells. Cultures of Ishikawa cells were divided into the following treatment groups: culture medium + DMSO (V, control group), 2ME + DMSO (2ME), culture medium + F-spondin antibody (anti-spon), F-spondin antibody + 2ME (anti-spon + 2ME). At 72 h after treatment, cultured cells were processed to measure their viability as described in the Materials and Methods section. Hydrogen peroxide (H_2_O_2_) was added as positive control of toxicity. This experiment consisted of 5 replicates. a ≠ b ≠ c ≠ d, *p* < 0.05.

This experiment was designed to establish if incubation with a neutralizing F-spondin antibody to Ishikawa cells could block the effect of 2ME on the cell viability. Ishikawa cells were incubated with 5 µM of 2ME alone or combined with F-spondin antibody 30 µg/mL, and 72 h later, the cells were processed to determine cell viability. Figure 6 shows that as expected, 2ME decreased cell viability at 72 h (33.0 ± 2.4% from the control group) while administration of F-spondin antibody blocked the effect of 2ME on the cell viability (68.3 ± 5.8% from the control group).

## 3. Discussion

The metabolite 2-methoxyestradiol induces apoptosis in a variety of human cancer cell lines. Herein, we show that F-spondin participates in the intracellular signaling pathway by which 2ME induces apoptosis in the Ishikawa cells. It has been shown that low pharmacological concentrations of 2ME did not trigger apoptosis in cancer cell lines while at higher concentrations this estrogen metabolite was able to induce death cell [12]. We have now found that the 2ME-target genes *Scd2* and *Snx6* respond to 2ME both in apoptotic or non-apoptotic concentrations, indicating that these genes are not involved in the anticancer effects of 2ME on human endometrial cells. It is known that *Scd2* and *Snx6* are associated with intracellular traffic of membrane receptors, lipid metabolism, and extracellular matrix remodeling [15,16,17]. Thus, it is probable that the role of *Scd2* and *Snx6* is associated with the effects of 2ME on uterine physiology. In this context, Sibonga et al. [18] showed that 2ME mimicked the effect of E_2_ on the endometrial proliferation and hypertrophy in ovariectomized rats. Further studies are necessary to elucidate the role of *Scd2* and *Snx6* on the effects of 2ME in the mammalian uterus.

As *Spon1* was only increased with the 2ME apoptotic concentration, this molecule could be a potential candidate to participate in the anticancer effect of 2ME on endometrial cells. It is recognized that F-spondin is a matricellular molecule that participates in the outgrowth of the neural tissue as well as in the inhibition of angiogenesis in the floor plate and paranotochordal area of the developing embryo (reviewed in [19]). Here we describe for the first time that F-spondin expression is regulated by the estrogen metabolite 2ME in the cancer cell line Ishikawa. Moreover, levels of *Spon1* and its encoded protein F-spondin were increased between 6 and 12 h after 2ME treatment. Since 2ME induces apoptosis in Ishikawa cells at 48 h of exposure [12] we assumed that F-spondin is an element of the signaling pathway by which 2ME exerts its apoptotic activity. 

Although 2ME can bind to the classical ER (ESR1 and ESR2) with a lower affinity compared with E_2_ and Ishikawa cells, both of which express ER, we found that 2ME increased the expression of the mRNA for F-spondin independent of the ER activation. These results reinforce the concept that F-spondin signaling could be associated with the apoptotic activity of 2ME on Ishikawa cells since both 2ME effects did not require a functional ER. In order to confirm this assertion, the effect of 2ME on *Spon1* expression in ER-negative Ishikawa cells should be investigated, but this was not done. Recently, a new ER denominated GPR30, which is a G-coupled receptor, has been reported [20]. This new ER participates in the nongenomic actions of E_2_ and it has been associated with the estrogen-related tumorogenesis and cardioprotection [21,22], and interestingly, deficiency of GPR30 and 2ME is related to preeclampsia occurrence [23]. Probably, 2ME induces *Spon1* expression via GPR30 activation in Ishikawa cells, but this remains to be demonstrated. Another possibility is the participation of a putative 2ME receptor, although identification of this kind of receptor is still controversial. 

We found that neutralization of F-spondin is able to block the apoptotic effect of 2ME in the Ishikawa cells, corroborating our proposal that 2ME induces apoptosis through activation of F-spondin signaling in the Ishikawa cells. Cellular interaction with the extracellular matrix is a requisite for cell proliferation, and for tumor growth, invasion, and metastasis [24]. In this context, integrin-dependent interaction with the extracellular matrix proteins plays an important role in the regulation of cancer cell proliferation, which is crucial for the growth of tumors and their ability to generate metastases [24]. Interestingly, it has been shown that F-spondin can block activity of integrin αvβ3, inhibiting angiogenesis in human umbilical vascular endothelial cells [25]. As integrin αvβ3 is expressed in endometrium cancer [26] it is feasible to propose that F-spondin inhibit proliferation in Ishikawa cells by interacting with the integrin αvβ3; however, this possibility was not explored in this study. To our knowledge, this is the first report relating a molecule with anti-angiogenic properties in the apoptotic effects of 2ME on uterine-derived cancers. Further studies are necessary to determine whether F-spondin can also mediate the apoptotic activity of 2ME in tumors of preclinical models, such as nude mice xenografted with Ishikawa cells or primary cultures of endometrial cancer cells from patients.

Activation of apoptosis in malignant cells is a promising anticancer therapy. The apoptotic effects of 2ME on endometrial cells cancer involve activation of the intrinsic (via p53, inactivation of Bcl-2 and accumulation of oxygen free radicals) as well as activation of the extrinsic (up-regulation of DR5) pathways [27,28]. Whether F-spondin activates some of these two apoptotic pathways needs to be determined. On the other hand, it is known that decreased levels of 2ME are associated with other uterine pathophysiological alterations including endometriosis and preeclampsia [29,30]. Interestingly, 2ME is able to induce apoptosis in endometriotic cells [31]. Thus, it is plausible to speculate that induction of apoptosis is a key mechanism by which 2ME and F-spondin signaling could exert their therapeutics properties. 

The evidence of numerous in vitro and in vivo tumor models indicates that 2ME is a promising anticancer agent. However, the potential therapeutic applications of 2ME in the anticancer strategy have been limited by its poor water solubility and low bioavailability, preventing it from reaching adequate plasma concentrations relative to its effective dose (reviewed in [2]). In this context, new biomarkers associated with the anticancer effects of 2ME, such as F-spondin, which respond to 2ME in primary cell cultures from endometrium cancer patients, could be used as a therapeutic strategy to enhance the probabilities of clinical management in patients in early stages of endometrial cancer. In addition, monitoring of F-spondin levels in the plasma of reproductive-aged women could be used as a marker to early cancer detection or to evaluate the efficacy of the 2ME treatment in patients. 

In summary, we conclude that F-spondin is one of the molecular signals by which 2ME induces apoptosis in Ishikawa cells. These results provide experimental evidence underlying the mechanism of action of 2ME on endometrial cancer cells.

## 4. Materials and Methods

### 4.1. Cell Culture

Ishikawa cells were maintained in DMEM/high modified medium with 4.0 mM L-glutamine and 4.500 mg/L glucose free of phenol red (Thermo Fisher Scientific, Waltham, USA) supplemented with 10% (*v*/*v*) of fetal bovine serum (Thermo Fisher Scientific), sodium pyruvate 1 mM, penicillin 100 UI/mL, and streptomycin 100 µg/mL (Sigma Chemical, St. Louis, MO, USA). The Ethical Committee of the Universidad de Santiago de Chile (DICYT 021743OD_DAS) approved this study. For all experiments, 1,000,000 Ishikawa cells/well were seeded.

### 4.2. Treatments

Previous works have already shown that a concentration of 2ME 0.1 µM is non-apoptotic while 2ME 5 µM is apoptotic to Ishikawa cells [12]. Cells were changed to medium without serum for 15 h before each treatment. Then, they were treated with 0 (control), 0.1, or 5 µM of 2ME (Sigma) dissolved in dimethyl sulfoxide (DMSO) 0.01% as vehicle. Other cultures were also incubated with the ER antagonist ICI182780 (25 µg/mL, Tocris Bioscience, Bristol, UK) or with the F-spondin neutralizing antibody (30 µg/mL, Catalog No. AF3135, R&D Systems, Inc., Minneapolis, MN, USA) dissolved in DMSO or PBS, respectively.

### 4.3. Measurement of Cell Viability

Cell viability was assessed by the MTS dye reduction assay using the CellTiter 96^®^ AQueous Non-Radioactive Cell Proliferation Assay kit (Promega, Madison, WI, USA) according to the manufacturer’s instructions. Briefly, Ishikawa cells were grown on 96-well assay plates and 72 h post-treatment, 20 µL of MTS reagent provided by the kit was added to each well. After incubation, the absorbance value at 490 nm was obtained using an ELISA plate reader (Tecan Group Ltd. Männedorf, Switzerland). As positive control of cytotoxicity, we added hydrogen peroxide (H_2_O_2_) 0.08% dissolved in 4 µL of culture medium.

### 4.4. Real-Time Polymerase Chain Reaction

Total RNA from Ishikawa cells was isolated using Trizol Reagent (Invitrogen, Carlsbad, NM, USA). One µg of total RNA of each sample was treated with DNase I Amplification grade (Invitrogen). The single-strand cDNA was synthesized by reverse transcription using the Superscript III Reverse Transcriptase First Strand System for RT-PCR (Invitrogen), according to the manufacturer’s protocol. A light cycler instrument (Roche Diagnostics, GmbH Mannheim, Germany) was used to quantify the relative mRNA level for *scd2*, *snx6*, or *spon1* in the Ishikawa cells; *Gapdh* was chosen as the housekeeping gene to be used as load control. SYBR^®^ Green I double-strand DNA binding dye (Roche Diagnostics) was used for these assays. Primers for *scd2* were 5’ CTTTGTCGCTGAGGTCTGAA 3´ (sense) and 5´ TTACTCAGCCACCACCACA 3´ (antisense); for *snx6* they were 5′ TAGAAGGTCTAGGTCGCTGGTAG 3′ (sense) and 5′ TGTGGTGGTGGCTGAGTAA 3′ (antisense); for *spon1* they were 5′ GAGAGATACGTGAAGCAGTTCC 3′ (sense) and 5′ ATACGGTGCCTCTTCTTCATAC 3′ (antisense); and for *Gapdh* they were 5′ TGCCAAATATGATGACATCAAGAA 3′ (sense) and 5′ GGAGTGGGTGTCGCTGTTG 3′ (anti sense). All real-time PCR assays were performed in duplicate. The thermal cycling conditions included an initial activation step at 95 °C for 25 min, followed by 40 cycles of denaturizing and annealing–amplification (95 °C for 15 s, 59 °C for 30 s, and 72 °C for 30 s), and finally one cycle of melting (95 to 60 °C). To verify specificity of the product, amplified products were subjected to melting curve analysis as well as electrophoresis, and product sequencing was performed to confirm identity using an ABI Prism310 sequencer (Applied Biosystems, Foster city, USA). The expression of *scd2, snx6*, and *spon1* were determined using the equation Y = 2^−∆*Cp*^ [32], where Y is the relative expression, Cp (crossing point) is the cycle in the amplification reaction in which fluorescence begins to be exponential above the background base line, and −∆Cp is the result of subtracting the Cp value of *Ar* from the Cp value of *gapdh* for each sample. The relative expression values were multiplied by 10^3^ to simplify the presentation of the data [32].

### 4.5. Immunoblotting

All the samples were processed in duplicate to determine the level of the F-spondin protein. Ishikawa cells were lysed in lysis buffer (20 mM Tris–HCl, pH 8.0, 137 mM NaCl, 1% Nonidet P-40, and 10% glycerol) supplemented with a protease inhibitor cocktail (Complete; Roche, Mannheim, Germany). The lysate was centrifuged at 4 °C for 10 min at 10,000*g* and the pellet was discarded. Protein concentrations in the supernatant were measured by the Bradford assay (Bio-Rad, Hercules, CA, USA). After boiling for 5 min, proteins (20 µg) were separated on 15% SDS-PAGE slab gels in a Mini PROTEAN electrophoretic chamber (Bio-Rad, Hercules, CA, USA). Proteins resolved in the gels were electroblotted onto nitrocellulose membranes (Bio-Rad). The membranes were blocked three hours in TTBS (100 mM Tris–HCl pH 7.5, 150 mM NaCl, and 0.05% *v*/*v* Tween-20) that contained 5% nonfat dry milk and were incubated overnight with rabbit anti-F-spondin1 (Santa Cruz Biotechnology, Santa Cruz, CA, USA) at 1:500 dilution. The immunoreactive band was visualized by incubation for 1 h with a secondary goat anti-rabbit IgG antibody (1:5000 dilution; Chemicon International, Temecula, CA) conjugated to horseradish peroxidase (HRP), followed by the Enhanced Western Lighting Chemiluminescence reaction (PerkinElmer Life Sciences, Boston, MA, USA). Blots were stripped and reprobed with rabbit anti β-actin antibody (1:5000 dilution, Sigma Chemical, St. Louis, MO, USA) and developed in a similar manner to ensure even loading. All blots were then digitalized and the relative level of F-spondin1 was normalized against β-actin. Negative controls consisting of blots without anti-F-spondin1 antibody were also included.

### 4.6. Immunofluorescence

Ishikawa cells were fixed in cold 4% paraformaldehyde in PBS pH 7.4–7.6 for 2 h, transferred to 10% *w/v* sucrose in PBS for 60 min at 4 °C, and 30% *w/v* sucrose in PBS at 4 °C overnight. Then, they were blocked with 1% PBS–BSA for 120 min, and incubated with 1:250 rabbit anti-F-spondin1 antibody (Santa Cruz Biotechnology, Santa Cruz, CA, USA). After washing with PBS, the preparations were incubated for 2 h with goat anti-rabbit IgG fluorescein isothiocyanate (FITC) conjugate (Santa Cruz Biotechnology) diluted 1:1000. Sections were washed and counterstained with 1 µg/mL of propidium iodide (Thermo Scientific, Rockford, IL, USA), washed again, and then mounted in Fluoromount G. As negative controls, the primary antibody was replaced by preimmune serum. All sections were visualized with an Optiphot Epifluoresence Microscope (Olympus, Middlebush, NJ, USA).

### 4.7. Statistical Analysis

All data were presented as mean ± SE. These data followed a non-normal distribution (Kolmogorov–Smirnov test) and significant differences between groups were determined through the use of variance analysis by Friedman’s test with subsequent post-hoc Wilcoxon signed-rank test. Significance was accepted at *p* < 0.05. Values for the experiments of cell viability were expressed as percentage from the control.
